# Adverse childhood experiences and adult self-harm in a female forensic population

**DOI:** 10.1192/bjb.2021.34

**Published:** 2022-06

**Authors:** Rachel Holden, Imogen Stables, Penelope Brown, Maria Fotiadou

**Affiliations:** 1 South London and Maudsley NHS Foundation Trust, UK; 2 King's College London, UK

**Keywords:** Trauma, forensic mental health services, in-patient treatment, self-harm, adverse childhood experiences

## Abstract

**Aims and method:**

This study aimed to investigate the prevalence of adverse childhood experiences (ACEs) among patients in a female forensic psychiatric in-patient medium-secure unit, and to analyse the link between ACEs, adulthood self-harm and associated comorbidities and risk factors. The study used a cross-sectional design, with data gathered from the anonymised electronic health records of patients.

**Results:**

It was found that there was a high prevalence of both ACEs and self-harm among this patient group, and that there was a relationship between the two; those with more ACEs were more likely to have self-harmed during adulthood. Of the individual ACE categories, it was also demonstrated that emotional abuse had a significant association with adulthood self-harm.

**Clinical implications:**

In medium-secure settings for women, implementation of trauma-informed care will be beneficial because of the high number of those with mental disorders who have experienced adversity during their childhood.

Adverse childhood experiences (ACEs) are stressful or traumatic life events that occur before 18 years of age.^1,2^ Having a history of ACEs is not uncommon: in a national household survey of adults residing in England, 47% of participants reported at least one ACE.^3^ Research on ACEs has demonstrated links between ACEs and self-harm.^4^ In a female prison population, all types of childhood abuse and neglect were more prevalent in those who self-harmed compared with those who did not, with significant associations between adulthood self-harm and both childhood emotional and sexual abuse.^5^ ACEs have a cumulative effect on health outcomes, with higher numbers of ACEs predicting more adverse health outcomes.^6^

This study occurred on Spring Ward, the female ward of River House Medium Secure Unit (MSU) in South London and Maudsley NHS Foundation Trust. Since opening in April 2008, the ward has offered a multidisciplinary biopsychosocial approach to support women toward recovery and reintegration into the community.^7^ In 2017, following the success of implementing the Healing Trauma programme (a gender-specific intervention for trauma victims),^8^ a trauma-informed care approach was introduced. Implementation involved team consultation, presentation and a training session on Stephanie Covington's Becoming Trauma-Informed Programme.^9^

This study was conducted as part of a service evaluation to determine the prevalence of ACEs in a female MSU cohort and to explore the relationship between ACEs and adulthood self-harm. Links between ACEs and adulthood self-harming behaviours, violence and comorbidities (such as personality disorder) will be explored, which will enable us to better understand the present and future needs of patients.

## Aims

Our first aim was to establish the prevalence of ACEs in patients in a female MSU, using a structured ACE questionnaire. Second, we aimed to establish the prevalence of adulthood self-harm, personality disorder, alcohol and drug misuse, and history of violence among female in-patients in the MSU. And finally, we aimed to explore the relationship between ACEs and adulthood self-harm.

## Method

### Design and procedure

The main study utilised a cross-sectional design, using medical records. The researchers conducted a thorough review of each patient's electronic medical records. Using previously recorded information, the amount of ACEs were calculated by the researcher, based upon the trauma history recorded in the patients records.

### Participants

All patients who were admitted to or were receiving continuing treatment on the ward between April 2008 and July 2019 were considered eligible for participation in this study. Criteria for admission to the ward include being over 18 years of age, committing an offence or having a significant history of violence. All patients are sectioned under the Mental Health Act 1983 (MHA) throughout their admission. The ward can accommodate 15 patients. Because of the relapsing and remitting nature of the mental disorders of many patients, several patients had multiple admissions during the study period; for these patients, data was gathered from medical records documented during their most recent admission.

Initially, 68 participants were included in the sample. Two participants were excluded from the analysis because of insufficient information in their medical records regarding their childhood, taking the final sample to 66 participants. Demographic and clinical information included age on admission, ethnicity, primary diagnosis, MHA section, personality disorder, alcohol and drug misuse, and violence history. Ethnicity was recorded into subsections: White British, Black British, Black African, Black Caribbean, Asian and other. Primary diagnosis was recorded into three categories, according to the ICD-10: schizophrenia spectrum disorders (codes F20–F29), mood [affective] disorders (codes F30–F39) and personality disorders (codes F60–F69).^10^ MHA sections of all patients were recorded into categories: forensic (section 37, sections 37 and 41, sections 47 and 49, and sections 48 and 49) and civil (section 3).^11^

### Data collection and analysis

Exposure to adverse experiences up to 18 years of age was assessed for each participant, following a thorough review of their medical notes; ACE history was summarised by a ten-item version of the Adverse Childhood Experiences Questionnaire.^12^ The ACE questionnaire consists of ten binary (yes/no) questions that assess exposure to emotional, physical and sexual abuse; emotional and physical neglect; and household dysfunction, including domestic violence, substance use and incarceration. Participants’ self-harm history since 18 years of age was obtained via medical records, along with comorbid personality disorder, alcohol and drug misuse, and history of violence. Self-harm was coded as a binary variable, with the presence of self-harm being recorded if there was any mention of self-harm or suicide attempt in adulthood mentioned in the medical records. ACEs were extracted from records of patients’ trauma history recorded in psychological and psychiatric reports contained in their medical records. Personality disorder presence was defined as a previous diagnosis of any type of personality disorder. Alcohol and drug misuse were defined as any positive history of problematic use of alcohol or drugs. History of violence was defined as any violence history before the event leading to admission. Each of these variables were recorded as dichotomous (yes/no) variables.

Data was analysed with the Statistical Package for Social Sciences (IBM SPSS, version 25 for Mac).

### Ethical approval

Ethical approval was granted by the Research, Outcomes and Service Evaluation Committee, a branch of the Behavioural and Developmental Psychiatry Clinical Academic Group of South London and Maudsley NHS Foundation Trust. The ethical approval included the use of anonymised medical records. This was a service development project using historic clinical records and as such the study was exempt from a need to provide informed consent.

## Results

Patients were aged between 18 and 72 years at admission (Table 1). The sample was primarily Black and minority ethnic, and most patients had a primary diagnosis of schizophrenia, schizotypal and delusional disorders (ICD-10 codes F20–F29).^10^ The majority of the sample had a history of drug misuse. Over a third of the sample had a history of alcohol misuse, and over a quarter had a comorbid personality disorder diagnosis.

**Table 1:** Demographic and clinical characteristics of the patient sample

Characteristic	Cases (*N* = 66)
Age at admission (years), mean (s.d.)	38.40 (±11.37)
Age categories (years), *n* (%)	
<25	10 (15.2)
25–34	13 (19.7)
35–44	26 (39.3)
45–54	12 (18.2)
≥55	5 (7.6)
Ethnicity, *n* (%)	
White British	20 (30.3)
Black British	13 (19.7)
Black African	13 (19.7)
Black Caribbean	10 (15.2)
Asian	2 (3.0)
Other	8 (12.1)
Primary diagnosis, *n* (%)	
F20–F29, Schizophrenia, schizotypal and delusional disorders	57 (86.4)
F30–F39, Mood [affective] disorders	4 (6.1)
F60–F69, Disorders of adult personality and behaviour	5 (7.6)
Comorbid personality disorder, *n* (%)	20 (30.3)
Alcohol misuse, *n* (%)	25 (37.9)
Drug misuse, *n* (%)	34 (51.5)

### Number and prevalence of ACEs

In the total sample, the mean number of ACEs was 2.89 (±2.35) (Table 2), with ACE number showing a positively skewed distribution. Within the sample, most individuals (*n* = 54, 81.9%) had experienced at least one ACE during childhood; of the total sample, 28.8% had experienced two to three ACEs and 37.9% had experienced four or more ACEs. Among the ACE categories, emotional and physical abuse were the most common, and the rarest ACE was incarceration of household members (Table 2).

**Table 2:** Main study prevalence of number of ACEs and each category of ACE

	Cases, *N* = 66
Number of ACEs, mean (s.d.)	2.89 (2.35)
Number of ACEs, *n* (%)	
0	12 (18.2)
1	10 (15.2)
2	14 (21.2)
3	5 (7.6)
4	6 (9.1)
5	7 (10.6)
6	7 (10.6)
7	4 (6.1)
8	0 (0.0)
9	1 (1.5)
10	0 (0.0)
ACE, *n* (%)	
Emotional abuse	27 (40.9)
Physical abuse	27 (40.9)
Sexual abuse	20 (30.3)
Emotional neglect	26 (39.4)
Physical neglect	20 (30.3)
Parental separation or divorce	26 (39.4)
Violence against mother	8 (12.1)
Household alcohol/drug misuse	13 (19.7)
Mental illness in household	21 (31.8)
Incarceration of household member	2 (3.0)

Within the sample, two was the most common amount of ACEs (56% of the sample experienced two or more ACEs) and emotional and physical abuse were the most common ACE categories. ACE, adverse childhood experience.

### Number of ACEs and adulthood self-harm

Adulthood self-harm in the sample was analysed. It was determined that over half of the sample had self-harmed during adulthood (*n* = 36, 54.5%). Because of the positive skewed nature of the number of ACEs, we ran a Spearman's correlation to assess the relationship between number of ACEs and adulthood self-harm on the total sample of 66 patients. There was a statistically significant positive correlation between number of ACEs and adulthood self-harm (*r*_s_(64) = 0.45, *P* < 0.001). Following this, binary logistic regression was performed to ascertain the effect of increasing number of ACEs on the likelihood of adulthood self-harm. The binary logistic regression model was statistically significant (*χ*^2^(1) = 15.11, *P* < 0.005). The model explained 27.4% (Nagelkerke *R*^2^) of the variance in adulthood self-harm, and correctly classified 75.7% of cases (specificity 83.3%, sensitivity 69.4%). For every one-point increase in number of ACEs, individuals were 1.62 times more likely to self-harm as adults.

### ACE categories and adulthood self-harm

We conducted an analysis to determine whether there was any association between individual ACE categories and adulthood self-harm. The total sample who had self-harmed during adulthood was analysed to determine the prevalence of each type of ACE within this category. In those who had self-harmed during adulthood, emotional abuse was the most common ACE (*n* = 22, 33.3%), followed by physical abuse (*n* = 19, 28.8%), emotional neglect (*n* = 19, 28.8%) and physical neglect (*n* = 17, 25.8%). Estimates of the odds ratio for each of the ten ACEs ranged from 1.38 (95% CI 0.47–3.99) for sexual abuse to 8.05 (95% CI 2.07–31.39) for physical neglect. However, statistically significant associations with adulthood self-harm were only seen for emotional abuse, physical abuse, emotional neglect and physical neglect. These relationships were then further explored by Pearson's *χ*^2^-tests and Fisher's exact tests, which confirmed that four categories of ACE had a statistically significant association with adulthood self-harm: emotional abuse (*χ*^2^(1) = 13.37, *P* < 0.001), physical abuse (*χ*^2^(1) = 4.62, *P* = 0.032), emotional neglect (*χ*^2^(1) = 5.94), *P* = 0.015) and physical neglect (*P* = 0.001, Fisher's exact test, two-sided).

Multivariate binary logistic regression was conducted to determine whether emotional abuse, physical abuse, emotional neglect and physical neglect maintained their statistically significant association with adulthood self-harm when all four categories were accounted for. In this model, 31.2% (Nagelkerke *R*^2^) of the variation in adulthood self-harm was explained by these four ACE categories. This model correctly classified 71.2% of cases (specificity 80.0%, sensitivity 63.9%). When accounting for emotional abuse, physical abuse, emotional neglect and physical neglect, only emotional abuse maintained a statistically significant relationship with adulthood self-harm (*P* = 0.034), showing that being emotionally abused increased the likelihood of adulthood self-harm by 7.36 times (Table 3).

**Table 3:** Multivariate binary logistic regression to analyse the association between emotional abuse, physical abuse, emotional neglect and physical neglect, and adulthood self-harm

	Variables	*B*	s.e.	Wald	d.f.	Significance	Exp(*B*)	95% CI for Exp(*B*)	
								Lower	Upper
Step 1	Emotional abuse	1.997	0.944	4.477	1	0.034	7.364	1.158	46.805
	Physical abuse	−0.507	0.905	0.314	1	0.575	0.602	0.102	3.549
	Emotional neglect	−0.965	1.116	0.748	1	0.387	0.381	0.043	3.395
	Physical neglect	1.927	1.138	2.868	1	0.090	6.868	0.739	63.859

This multivariate binary logistic regression model included all adverse childhood experience categories that were individually significantly associated with adulthood self-harm. It was run to determine whether statistical significance was maintained when all the categories were accounted for; only emotional abuse remained significantly associated with adulthood self-harm.

## Discussion

This study was the first to analyse the prevalence of ACEs, adulthood self-harm and their relationship in a female MSU population in the UK. Multivariate binary logistic regression revealed a statistically significant association between an increasing number of ACEs and increased likelihood of adulthood-self harm. Emotional abuse was shown to have a statistically significant association with adulthood self-harm.

We found a high prevalence of ACE exposure in this female MSU cohort, with over 80% of individuals experiencing at least one ACE and 56% experiencing more than two ACEs. The prevalence of ACE exposure among this female MSU group was higher than the 47% prevalence in the general adult population of the UK.^3^

Within this population, there was a high prevalence of adulthood self-harm. Adulthood self-harm in our sample was reported by 54.5%, similar to the rates reported by Ribeiro et al^13^ (whose MSU population overlapped with ours), who found that 46.7% had a documented history of self-harm before MSU admission. Baker et al^14^ interviewed female patients in a medium-secure setting and discussed their experiences of self-harm; an overarching theme discussed was that of ‘the traumatised individual’, suggesting that individuals linking their traumatic experiences to self-harming behaviour is not uncommon. The high prevalence of ACEs amongst the female MSU population indicates that a trauma-informed approach to care in MSU settings for women is crucial. Application of ‘universal trauma precautions’ is necessary, to ensure that all who have been exposed to ACEs receive care that is not only growth-promoting, but also less likely to cause re-traumatisation than standard care.^15^

We found a statistically significant correlation between an increasing number of ACEs and the likelihood of adulthood self-harm. This is similar to research by Cleare et al,^16^ showing that those with a history of repeat self-harm were significantly more likely to report exposure to multiple ACEs. Moreover, our finding of a statistically significant relationship between emotional abuse and adulthood self-harm supports the research by Howard et al,^5^ who found a statistically significant association between emotional abuse and self-harm in a sample of female prisoners.

It was advantageous to focus on those admitted to a single female MSU over the past 11 years, as there is limited data regarding this population. Use of electronic medical records to obtain data meant minimal information was missing and there was low attrition. In this niche population, we achieved a good sample size; only around 12% of the 3500 MSU beds in the UK are occupied by women.^17^

The ACE questionnaire is limited as it provides no information regarding the severity, degree, duration, timing or quality of each ACE component, which may differ significantly from person to person. Furthermore, data collected about self-harm behaviours could have been improved by using the Inventory of Statements about Self-Injury questionnaire,^18^ examining the type, frequency, severity and reasons for the behaviour. Further research could focus on collecting more in-depth childhood histories from patients, or using self-harm measures that capture frequency and severity.

Limited research is also available in forensic psychiatric settings, specifically MSUs, and nationwide research into ACEs and self-harm within these units could be beneficial. Furthermore, the neurodevelopmental and psychological mechanisms by which ACEs and self-harm are linked need exploration.

## Data Availability

The data that support the findings of this study are available from the corresponding author, R.H., upon reasonable request.

## About the authors

**Rachel Holden** is a clinical psychologist at South London and Maudsley NHS Foundation Trust, UK, and a Clinical Psychologist Lecturer with the Institute of Psychiatry, Psychology & Neuroscience at King's College London, UK. **Imogen Stables** is a Medical Student at GKT School of Medical Education, King's College London, UK. **Penelope Brown** is a Consultant Forensic Psychiatrist at South London and Maudsley NHS Foundation Trust, UK. **Maria Fotiadou** is a Consultant Forensic Psychiatrist South London and Maudsley NHS Foundation Trust, UK.
